# RACIAL/ETHNIC AND SEX DIFFERENCES IN LATE-LIFE COGNITIVE TRAJECTORIES

**DOI:** 10.1093/geroni/igae098.2184

**Published:** 2024-12-31

**Authors:** Olga Jarrín, Roland Thorpe, Fred Kobylarz, Weiyi Xia, Hyosin Kim, Anum Zafar, Maria Lopez, Haiqun Lin

**Affiliations:** Rutgers, The State University of New Jersey, New Brunswick, New Jersey, United States; Johns Hopkins University, Baltimore, Maryland, United States; Rutgers Robert Wood Johnson Medical School, New Brunswick, New Jersey, United States; Rutgers, The State University of New Jersey, Piscataway, New Jersey, United States; Oregon State University, Corvallis, Oregon, United States; Rutgers, The State University of New Jersey, New Brunswick, New Jersey, United States; Rutgers, The State University of New Jersey, New Brunswick, New Jersey, United States; Rutgers, The State University of New Jersey, Newark, New Jersey, United States

## Abstract

Prior research on racial/ethnic and sex difference in late life cognitive trajectories has been limited by small sample sizes in clinical and survey cohorts. We overcome these challenges through leveraging real-world data for the U.S. Medicare beneficiary population to examine within-group differences in cognitive function trajectories and their predictors. Specifically, we found Black, Hispanic, and Asian American men and women have a higher risk of severe cognitive impairment and spend more months and years of their lives with significant cognitive impairment. These differences are largely driven by differences in the risk of cognitive impairment associated with older age, consistent with prior research on life course stress and the ‘weathering’ hypothesis that early health deterioration is a consequence of the cumulative impacts of social or economic adversity and political marginalization. Our findings replicate, and in some cases refute, findings from prior studies from smaller longitudinal cohorts, including the Health and Retirement Study. Results will be discussed from a life course perspective with attention to variation in environmental, sociocultural, political, and historical factors that create and sustain health disparities for racial/ethnic minority older adults in the United States.

